# TBK1 recruitment to STING mediates autoinflammatory arthritis caused by defective DNA clearance

**DOI:** 10.1084/jem.20211539

**Published:** 2021-12-13

**Authors:** Tong Li, Seoyun Yum, Minghao Li, Xiang Chen, Xiaoxia Zuo, Zhijian J. Chen

**Affiliations:** 1 Department of Molecular Biology, University of Texas Southwestern Medical Center, Dallas, TX; 2 Center for Inflammation Research, University of Texas Southwestern Medical Center, Dallas, TX; 3 Department of Rheumatology and Immunology, Xiangya Hospital, Central South University, Changsha, Hunan, China; 4 Howard Hughes Medical Institute, Chevy Chase, MD

## Abstract

Defective DNA clearance in *DNase II*^−/−^ mice leads to lethal inflammatory diseases that can be rescued by deleting cGAS or STING, but the role of distinct signaling pathways downstream of STING in the disease manifestation is not known. We found that the STING S365A mutation, which abrogates IRF3 binding and type I interferon induction, rescued the embryonic lethality of *DNase II*^−/−^ mice. However, the STING S365A mutant retains the ability to recruit TBK1 and activate NF-κB, and *DNase II*^−/−^ *STING*-S365A mice exhibited severe polyarthritis, which was alleviated by neutralizing antibodies against TNF-α or IL-6 receptor. In contrast, the STING L373A mutation or C-terminal tail truncation, which disrupts TBK1 binding and therefore prevents activation of both IRF3 and NF-κB, completely rescued the phenotypes of *DNase II*^−/−^ mice. These results demonstrate that TBK1 recruitment to STING mediates autoinflammatory arthritis independently of type I interferons. Inhibiting TBK1 binding to STING may be a therapeutic strategy for certain autoinflammatory diseases instigated by self-DNA.

## Teaser

Using mice engineered to exhibit specific defects in distinct signaling pathways downstream of STING, Li et al. demonstrate that TBK1, but not IRF3, recruitment to STING mediates autoinflammatory arthritis caused by DNase II deficiency.

## Introduction

Autoimmune and inflammatory diseases, including systemic lupus erythematosus (SLE) and rheumatoid arthritis (RA), are characterized by the loss of immune self-tolerance and subsequent multiorgan inflammation and destruction. Although the causes of autoimmune diseases are often complex and involve multiple genetic and environmental factors, the accumulation of DNA from dying cells and defective DNA clearance are considered critical factors that contribute to the disease pathogenesis (Mahajan et al., 2016). Consistent with this idea, mutations in DNases were found to be associated with SLE and other autoimmune diseases (Keyel, 2017).

Among DNase-deficient mouse models, *DNase II*^−/−^ mice show embryonic lethality accompanied by inflammation and anemia (Kawane et al., 2001). DNase II is an endonuclease in the lysosome, where it degrades exogenous DNA engulfed by macrophages. During development, DNase II–deficient macrophages fail to digest nuclear DNA expelled from erythroid precursor cells, and the subsequent DNA accumulation causes inflammation and embryonic lethality (Kawane et al., 2001). For SLE patients, DNase II polymorphisms were associated with a risk of renal disorder (Shin et al., 2005). In addition, biallelic loss-of-function mutations in the DNase II gene were found in patients displaying severe neonatal anemia, glomerulonephritis, liver fibrosis, and deforming arthropathy (Rodero et al., 2017).

Type I IFN production is responsible for the lethality of *DNase II*^−/−^ mice; depleting the type I IFN receptor 1 (IFNAR1) allowed *DNase II*^−/−^ mice to survive to adulthood (Kawane et al., 2006; Yoshida et al., 2005). However, *DNase II*^−/−^ *Ifnar1*^−/−^ mice developed chronic polyarthritis that resembles RA (Kawane et al., 2006). Inflammatory cytokines such as TNF-α, IL-1β, and IL-6 were major drivers of this polyarthritis (Kawane et al., 2006; Kawane et al., 2010). Strikingly, ablating members of the cytosolic DNA-sensing pathway such as cyclic GMP-AMP synthase (cGAS) or stimulator of IFN genes (STING) rescued *DNase II*^−/−^ mice from both embryonic lethality and inflammatory arthritis (Ahn et al., 2012; Gao et al., 2015).

cGAS is a DNA sensor that detects double-stranded DNA invading the cytosol of a cell (Sun et al., 2013; Wu et al., 2013). cGAS binds DNA in a sequence-independent manner, allowing the detection of not only microbial DNA but also self-DNA that arises from cellular damage or defective DNA clearance (Ablasser et al., 2014; Ahn et al., 2012; Gall et al., 2012; Gao et al., 2015; Gray et al., 2015). Upon DNA binding, cGAS synthesizes a second messenger cyclic GMP-AMP (cGAMP) that binds to the downstream adaptor protein STING on the ER membrane (Ishikawa and Barber, 2008; Sun et al., 2009; Wu et al., 2013; Zhong et al., 2008). Activated STING proteins oligomerize and recruit TANK-binding kinase 1 (TBK1) to the [(D or E)xPxPLR(S or T)D] motif (in which “x” denotes any amino acid) at the C-terminal tail (CTT) of STING (Zhang et al., 2019). TBK1 phosphorylates the CTT of a neighboring STING, including the serine residue within the IFN regulatory factor 3 (IRF3) binding [pLxIS] motif (in which “p” represents a hydrophilic residue), thereby recruiting IRF3 to the phosphorylated STING (Liu et al., 2015; Tanaka and Chen, 2012; Zhang et al., 2019). Using STING as a scaffold, TBK1 then phosphorylates IRF3, which translocates to the nucleus to induce type I IFNs (Ishikawa and Barber, 2008; Sun et al., 2009; Zhong et al., 2008). TBK1 and its homologue IκB kinase ε (IKKε) also lead to activation of the IKK complex, which then activates the transcription factor NF-κB (Abe and Barber, 2014; Balka et al., 2020). NF-κB promotes IFN production by synergizing with IRF3 and also induces proinflammatory cytokines independently of IRF3. In addition, STING induces noncanonical autophagy to clear DNA or pathogens from the cytosol (Gui et al., 2019).

Inflammatory arthritis observed in *DNase II*^−/−^ *Ifnar1*^−/−^ mice (Kawane et al., 2006), but not in *DNase II*^−/−^ *Sting*^−/−^ mice (Ahn et al., 2012) or *DNase II*^−/−^ *cGas*^−/−^ mice (Gao et al., 2015), indicates that STING activation by self-DNA is responsible for the inflammatory cytokine production. Although NF-κB activation was suggested to induce inflammatory cytokines in vitro, the role of STING-induced cytokines by self-DNA in vivo remains elusive. We recently generated STING mutant mice with distinct signaling defects: S365A, L373A, and CTT truncation (Yum et al., 2021). The *STING*-S365A mutation disrupts the IRF3 binding site and thus selectively ablates IFN production while retaining TBK1 binding and NF-κB activation, as well as the ability to induce autophagy. The *STING*-ΔCTT and *STING*-L373A mutations disrupt the TBK1 binding site and are predicted to also disrupt IKKε binding based on sequence homology, although this remains to be tested. Cells harboring the STING-ΔCTT and STING-L373A mutations lacked phosphorylation of TBK1 and IKKε and the subsequent activation of IRF3 and NF-κB in response to stimulation (Balka et al., 2020; Yum et al., 2021).

To determine the molecular mechanism of inflammatory arthritis caused by defects in DNA clearance, we generated *DNase II*^−/−^ *STING* mutant (S365A, L373A, or ΔCTT) mice. Here we show that all *DNase II*^−/−^ *STING* mutant mice were rescued from lethality and survived into adulthood. However, *DNase II*^−/−^ *STING*^S365A/S365A^ mice, but not *STING*^ΔCTT/ΔCTT^ or *STING*^L373A/L373A^ mice, still displayed polyarthritis, footpad inflammation, and elevated levels of proinflammatory cytokines. Blocking TNF-α or IL-6 signaling using neutralizing antibodies alleviated arthritis manifestations in *DNase II*^−/−^ *STING*^S365A/S365A^ mice. These results demonstrate that the recruitment of TBK1 but not IRF3 to STING is essential for the inflammatory polyarthritis caused by defective DNA clearance.

## Results

### *DNase II*^−/−^ *STING*^S365A/S365A^ mice, but not *DNase II*^−/−^ *STING*^ΔCTT/ΔCTT^ or *DNase II*^−/−^ *STING*^L373A/L373A^ mice, develop polyarthritis

**Figure 1.**
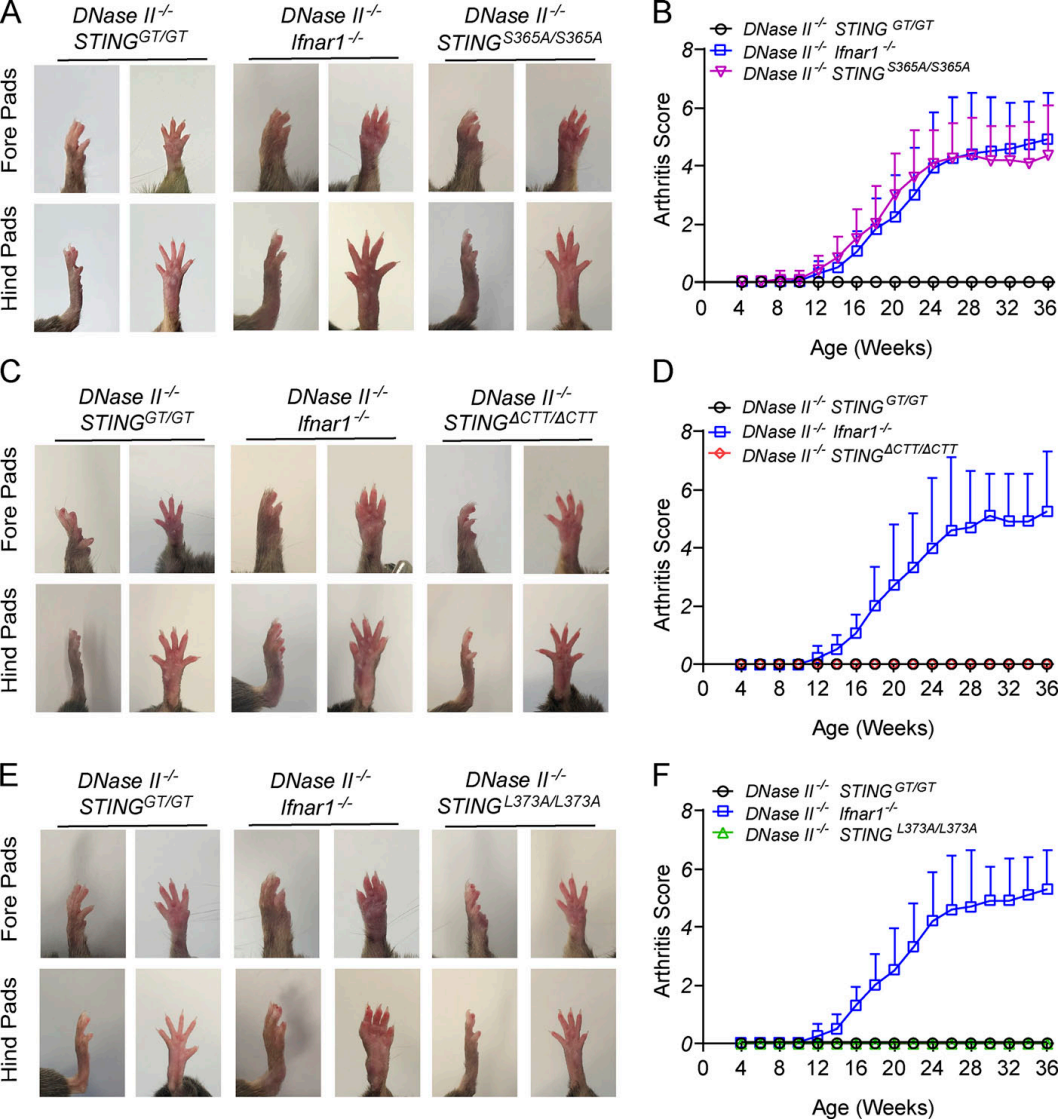
**TBK1 recruitment to STING is required for polyarthritis in *DNase II*^−/−^ mice. (A, C, and E)** Representative images of 8-mo-old male mouse footpads. **(B, D, and F)** Arthritis scores of the forelimb and hindlimb (*n* = 10–12 in each genotype). Results are representative of two independent experiments.

While *DNase II*^−/−^ mice are embryonically lethal (Kawane et al., 2001), all *DNase II*^−/−^ mice harboring STING mutations, including S365A, L373A, ΔCTT, or Goldenticket (gt; which lacks STING expression; Sauer et al., 2011), were born normally, similar to *DNase II*^−/−^ *Ifnar1*^−/−^ mice. However, *DNase II*^−/−^ *Ifnar1*^−/−^ and *DNase II*^−/−^*STING*^S365A/S365A^ mice still developed polyarthritis characterized by swelling of footpads starting from 10–12 wk of age (Fig. 1, A and B). In contrast, *DNase II*^−/−^ *STING*^ΔCTT/ΔCTT^ mice were completely rescued from the polyarthritis (Fig. 1, C and D), suggesting that the interaction of STING with downstream mediators through the CTT is required for the disease phenotypes. Similarly, *DNase II*^−/−^ *STING^L^*^373A/L373A^ mice (Fig. 1, E and F) did not manifest any signs of arthritis, specifically indicating that TBK1 recruitment to STING mediates the arthritis development caused by defective DNA clearance. No apparent sex difference in phenotypes was observed for all mice used in this study.

### TBK1 recruitment to STING causes inflammation in footpads of *DNase II*^−/−^ mice

**Figure 2.**
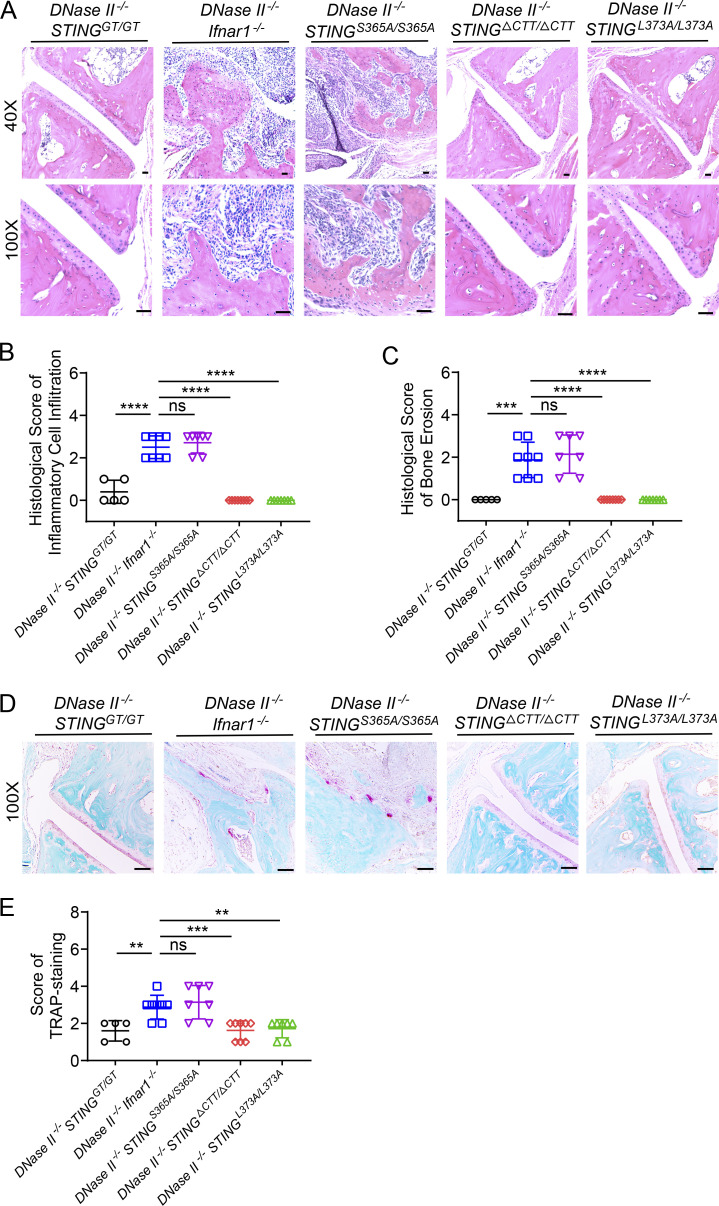
**TBK1 recruitment to STING induces inflammation and bone erosion in *DNase II*^−/−^ mouse footpads. (A–C)** H&E-stained joint tissues from hind footpads of 8-mo-old male mice (*n* = 5–8 in each genotype). **(A)** Representative images. Scale bars, 200 µm. **(B and C)** Blinded analyses of histologic scores calculated as described in Materials and methods (mean ± SD, *n* = 5–8 per genotype). **(D)** Representative images of TRAP-stained joint sections from 8-mo-old male mice. Scale bars, 200 µm. **(E)** TRAP scores calculated as described in Materials and methods (mean ± SD, *n* = 5–8 per genotype). For B, C, and E, each dot represents an individual mouse. Statistical analysis was done using a two-tailed, unpaired Student’s test. **, P < 0.01; ***, P < 0.001; ****, P < 0.0001. The histologic scores were evaluated separately by two pathologists.

H&E staining of footpads of *DNase II*^−/−^ *Ifnar1*^−/−^ and *DNase II*^−/−^ *STING*^S365A/S365A^ mice confirmed aggressive synovitis and bone erosion (Fig. 2, A–C). In addition, more osteoclasts were found in the joints of *DNase II*^−/−^ *STING*^S365A/S365A^ mice, indicated by the staining of tartrate-resistant phosphatase (TRAP; Fig. 2, D and E). Infiltration of inflammatory cells, bone erosion, and osteoclast accumulation did not occur in *DNase II*^−/−^ *STING*^GT/GT^, *STING*^ΔCTT/ΔCTT^, or *STING*^L373A/L373A^ mice (Fig. 2, A–E). These data indicate that TBK1 recruitment to STING mediates inflammation of *DNase II*^−/−^ mice.

### TBK1 recruitment to STING induces inflammatory cytokines and increases blood monocytes in *DNase II*^−/−^ mice

**Figure 3.**
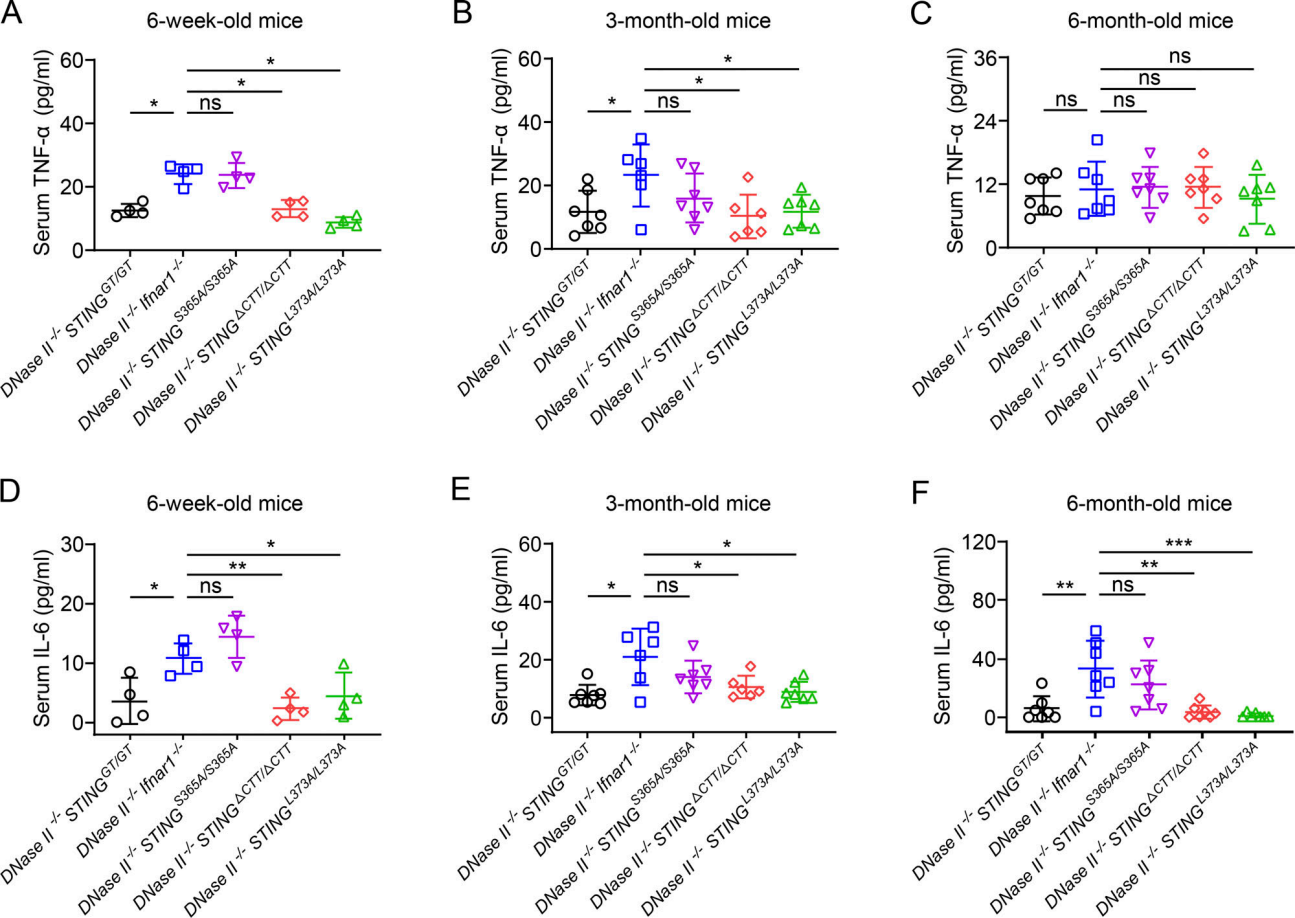
**TBK1 recruitment to STING elevates serum TNF-α and IL-6 levels in *DNase II*^−/−^ mice. (A–C)** TNF-α levels in the serum of 6-wk-old male (A), 3-mo-old female (B), and 6-mo-old female (C) mice of the indicated genotypes (mean ± SD, *n* = 4–7 per genotype). **(D–F)** IL-6 levels in the serum of 6-wk-old male (D), 3-mo-old female (E), and 6-mo-old female (F) mice of the indicated genotypes (mean ± SD, *n* = 4–7 per genotype). Each dot represents an individual mouse. Statistical analysis was done using a two-tailed, unpaired Student’s test. *, P < 0.05; **, P < 0.01; ***, P < 0.001. Results are representative of two independent experiments.

TNF-α and IL-6 are key cytokines that drive inflammation in arthritis; TNF-α levels were elevated in patients with loss-of-function mutations in the DNase II gene (Rodero et al., 2017), and both TNFα and IL-6 levels were high in *DNase II*^−/−^ *Ifnar1*^−/−^ mice (Kawane et al., 2006; Kawane et al., 2010). We detected systemic production of TNF-α and IL-6 in the sera of 6-wk-old *DNase II*^−/−^ *Ifnar1*^−/−^ and *DNase II*^−/−^ *STING*^S365A/S365A^ mice before the development of arthritis (Fig. 3, A and D). This early production of STING-induced TNF-α and IL-6 was completely abrogated in *DNase II*^−/−^ *STING*^ΔCTT/ΔCTT^ and *DNase II*^−/−^ *STING*^L373A/L373A^ mice (Fig. 3, A and D). The levels of serum TNF-α in *DNase II*^−/−^ *Ifnar1*^−/−^ and *DNase II*^−/−^ *STING*^S365A/S365A^ mice gradually decreased in 3-mo-old (Fig. 3 B) and 6-mo-old (Fig. 3 C) mice, whereas the level of serum IL-6 gradually increased in older mice (Fig. 3, E and F). The kinetics of TNF-α and IL-6 production in these mice is consistent with previous reports suggesting that macrophages and synovial fibroblasts are the major sources of TNF-α and IL-6, respectively (Kinne et al., 2000; Yoshitomi, 2019).

**Figure 4.**
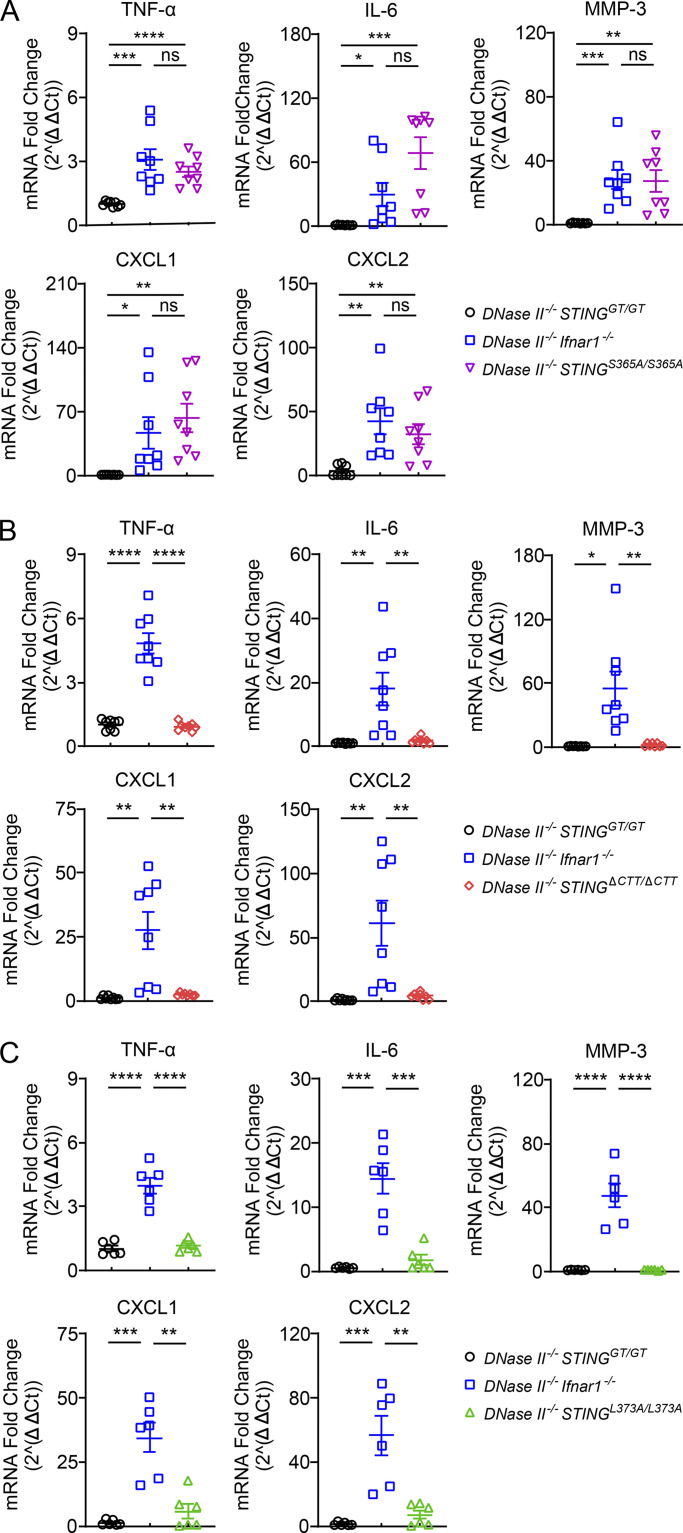
**TBK1 recruitment to STING induces inflammatory cytokines in *DNase II*^−/−^ mouse paws. (A–C)** Quantitative RT-PCR analysis of inflammatory cytokine levels in paws from 8-mo-old male mice of the indicated genotypes (mean ± SD, *n* = 6–8 per genotype). Each dot represents a mouse. Statistical analysis was done using a two-tailed, unpaired Student’s test. *, P < 0.05; **, P < 0.01; ***, P < 0.001; ****, P < 0.0001. Results are representative of four independent experiments. Ct, cycle threshold.

In addition to TNF-α and IL-6 that induce inflammation in the joints, matrix metallopeptidase 3 (MMP-3) degrades the extracellular matrix to drive bone erosion and is used as a marker of RA activity (Green et al., 2003; Tchetverikov et al., 2003). The expression of TNF-α, IL-6, and MMP-3 were all up-regulated in the paws of 8-mo-old *DNase II*^−/−^ *Ifnar1*^−/−^ and *DNase II*^−/−^ *STING*^S365A/S365A^ mice but not in the paws of *DNase II*^−/−^ *STING*^ΔCTT/ΔCTT^ and *DNase II*^−/−^ *STING*^L373A/L373A^ mice (Fig. 4, A–C). Our previous transcriptome analysis of bone marrow–derived macrophages showed that activation of STING-S365A induces high levels of C-X-C motif chemokine ligand 1 (CXCL1) and CXCL2 (Yum et al., 2021), which can recruit monocytes and neutrophils to the site of inflammation. Inflamed mouse paws from *DNase II*^−/−^ *Ifnar1*^−/−^ and *DNase II*^−/−^ *STING*^S365A/S365A^ mice, but not from other STING mutant mice, expressed high levels of CXCL1 and CXCL2 (Fig. 4, A–C).

**Figure 5.**
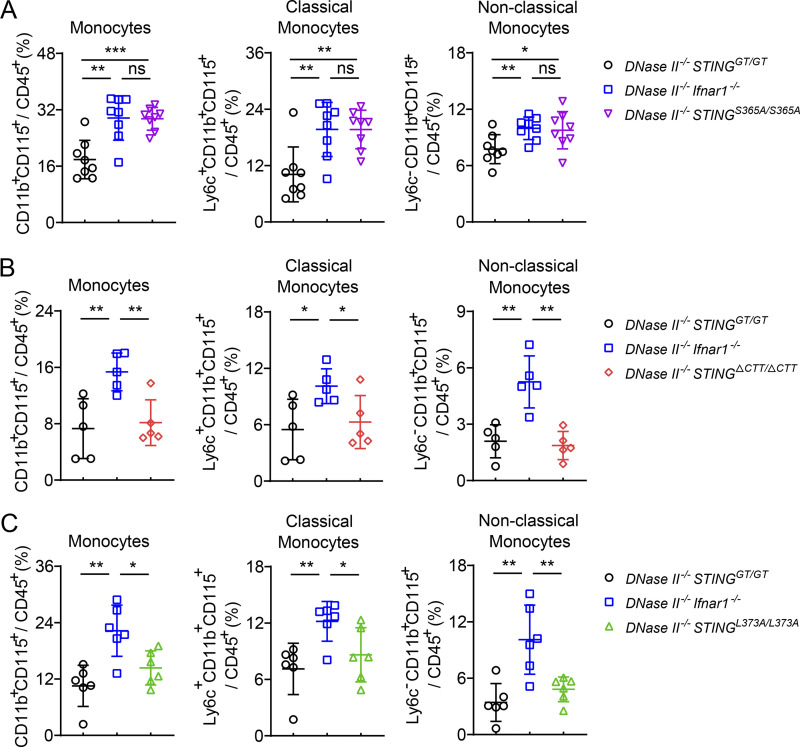
**TBK1 recruitment to STING increases blood monocyte populations in *DNase II*^−/−^ mice. (A–C)** FACS analysis of monocytes in the blood from 2–3-mo-old female mice of the indicated genotypes (mean ± SD, *n* = 5–8 in each genotype). Each dot represents a mouse. Statistical analysis was done using a two-tailed, unpaired Student’s test. *, P < 0.05; **, P < 0.01; ***, P < 0.001. Results are representative of two independent experiments.

Blood circulating monocytes are recruited into the RA synovium and contribute to inflammatory and destructive processes of the synovial joints (Rana et al., 2018). Furthermore, nonclassic monocytes are the precursors of macrophages that drive inflammation and bone erosion in different inflammatory arthritis models (Misharin et al., 2014). We observed increased numbers of classic monocytes (Ly6c^+^) and nonclassic monocytes (Ly6c^−^) in *DNase II*^−/−^ *Ifnar1*^−/−^ and *DNase II*^−/−^ *STING*^S365A/S365A^ mice compared to *DNase II*^−/−^ *STING*^GT/GT^ mice (Fig. 5 A). These circulating monocyte levels were significantly decreased in *DNase II*^−/−^ *STING*^ΔCTT/ΔCTT^ and *DNase II*^−/−^ *STING*^L373A/L373A^ mice (Fig. 5, B and C), suggesting that TBK1 recruitment to STING induces circulating monocytes that may lead to increased inflammatory cells and cytokines in the joints.

### TNF-α and IL-6 mediate inflammatory arthritis in *DNase II*^−/−^ *STING*^S365A/S365A^ mice

**Figure 6.**
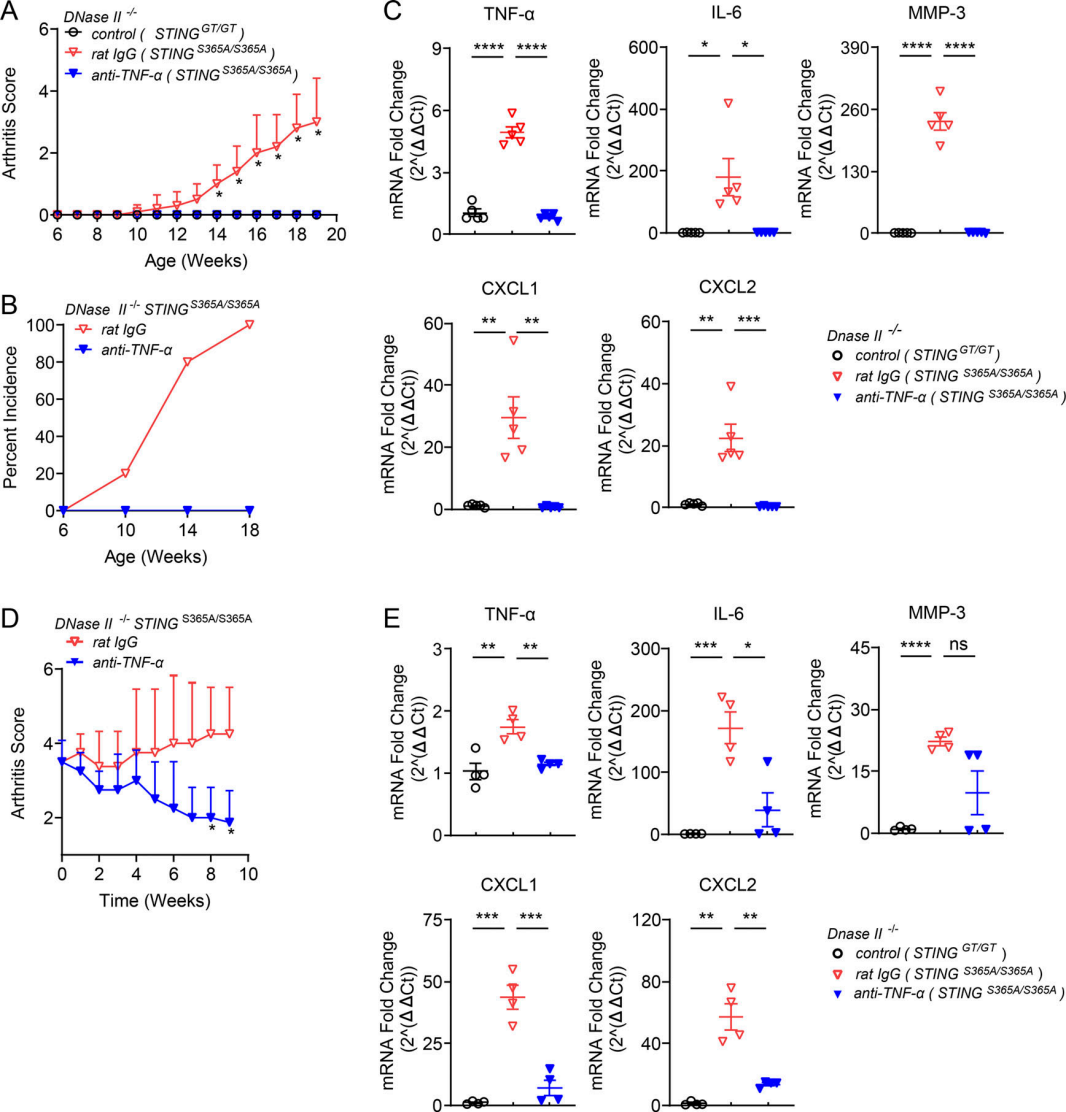
**Blocking TNF-α prevents the development of inflammatory arthritis in *DNase II*^−/−^ *STING*^S365A/S365A^ mice. (A–C)** 6-wk-old *DNase II*^−/−^ *STING*^S365A/S365A^ female mice received rat IgG or anti–TNF-α (40 µg/g body weight) i.p. once a week (*n* = 5 in each treatment group). **(A)** Arthritis scores were assessed every week (mean ± SD, *n* = 5 per group). Statistical analysis was done using a two-tailed, unpaired Student’s test. *, P < 0.05 between anti–TNF-α– and rat IgG–treated mice. **(B)** Incidence rates of arthritis (mean ± SD, *n* = 5 in each group). **(C)** qRT-PCR analysis of inflammatory cytokines in mouse paws (mean ± SD, *n* = 5 in each group). Each dot represents a mouse. **(D and E)** 5-mo-old male *DNase II*^−/−^ *STING*^S365A/S365A^ mice displaying polyarthritis received rat IgG or anti–TNF-α (20 µg/g body weight) twice a week (*n* = 4 in each group). **(D)** Arthritis scores were assessed weekly (mean ± SD, *n* = 4 per group). *, P < 0.05 between rat IgG– and anti–TNF-α–treated mice. **(E)** qRT-PCR analysis of inflammatory cytokines in mouse paws (mean ± SD, *n* = 4 per group). Statistical analysis was done using a two-tailed, unpaired Student’s test. *, P < 0.05; **, P < 0.01; ***, P < 0.001; ****, P < 0.0001. Results are representative of two independent experiments. Ct, cycle threshold.

Various cytokines are abundant in RA tissues, and TNF-α was shown to drive the production of other cytokines to induce inflammation (Feldmann et al., 1996). Blocking TNF-α provided both protective and therapeutic effects against arthritis in *DNase II*^−/−^ *Ifnar1*^−/−^ mice (Kawane et al., 2006). To determine if TNF-α is responsible for developing inflammatory arthritis in *DNase II*^−/−^ *STING*^S365A/S365A^ mice, we injected neutralizing antibodies against TNFα (anti–TNF-α) into mice once a week starting from 6 wk of age. The control IgG–treated group developed polyarthritis after 10 wk of age, but the anti–TNF-α–treated group was protected from arthritis development (Fig. 6, A and B). Blocking TNF-α also abrogated the induction of TNF-α, IL-6, MMP-3, CXCL1, and CXCL2 in mouse paws (Fig. 6 C), suggesting that TNF-α drives inflammatory gene expression in *DNase II*^−/−^ *STING*^S365A/S365A^ mice.

To evaluate the therapeutic effect of TNF-α blockade, we treated 5-mo-old *DNase II*^−/−^ *STING*^S365A/S365A^ mice that had already developed arthritis with anti–TNF-α (Fig. 6, D and E). Importantly, blocking TNF-α alleviated arthritis (Fig. 6 D) and greatly reduced the expression levels of inflammatory genes (Fig. 6 E).

**Figure 7.**
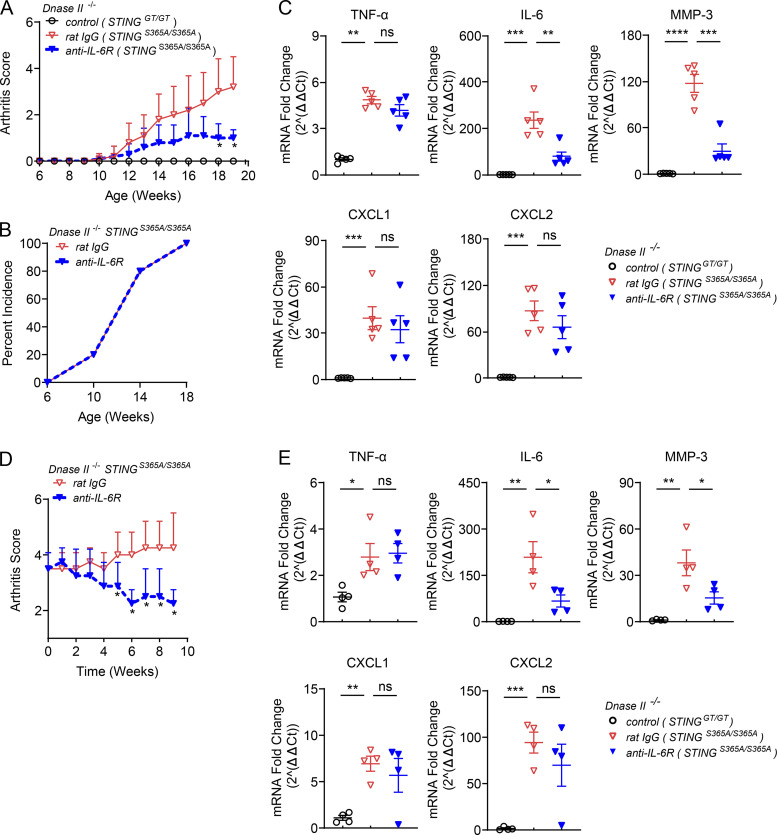
**Blocking IL-6R alleviates polyarthritis and joint inflammation in *DNase II*^−/−^ *STING*^S365A/S365A^ mice. (A–C)** 6-wk-old *DNase II***^−/−^** *STING*^S365A/S365A^ female mice received rat IgG or anti–IL-6R (100 µg/g body weight) once i.v., followed by i.p. treatment of antibodies (25 µg/g body weight) once a week (*n* = 5 in each treatment group). **(A)** Arthritis scores (mean ± SD, *n* = 5 per group). Statistical analysis was done using a two-tailed, unpaired Student’s test. *, P < 0.05 between rat IgG– and anti–IL-6R–treated mice. **(B)** Incidence rates of arthritis (*n* = 5 per group). **(C)** qRT-PCR analysis of inflammatory cytokines in mouse paws (mean ± SD, *n* = 5 in each group). Each dot represents a mouse. **(D and E)** 5-mo-old male *DNase II***^−/−^** *STING*^S365A/S365A^ mice displaying polyarthritis received rat IgG or anti–IL-6R (100 µg/g body weight) once, followed by i.p. treatment of antibodies (25 µg/g body weight) once a week (*n* = 4 in each group). **(D)** Arthritis scores. *, P < 0.05 between rat IgG– and anti–IL-6R–treated mice. **(E)** qRT-PCR analysis of inflammatory cytokines in mouse paws (mean ± SD, *n* = 4 per group). Statistical analysis was done using a two-tailed, unpaired Student’s test. *, P < 0.05; **, P < 0.01; ***, P < 0.001; ****, P < 0.0001. Results are representative of two independent experiments. Ct, cycle threshold.

IL-6 is a mediator of multiple inflammatory diseases (Kishimoto, 2005), including arthritis in DNase II–deficient mice (Kawane et al., 2010). To determine the role of IL-6 in the development of arthritis, we treated 6-wk-old *DNase II*^−/−^ *STING*^S365A/S365A^ mice with a neutralizing antibody against the IL-6 receptor (anti–IL-6R) once a week. Blocking IL-6 signaling reduced arthritis scores (Fig. 7 A) but did not block the incidence of arthritis (Fig. 7 B). The expression levels of IL-6 and MMP-3 in mouse paws were reduced by anti–IL-6R treatment; however, levels of TNF-α, CXCL1, and CXCL2 remained unchanged (Fig. 7 C). Treating the 5-mo-old *DNase II*^−/−^ *STING*^S365A/S365A^ mice with anti–IL-6R reduced the arthritis score (Fig. 7 D) and the expression of IL-6 and MMP-3 but did not reduce the expression of TNF-α, CXCL1, and CXCL2 (Fig. 7 E).

Altogether, these results demonstrate a critical role for TNF-α and IL-6 in driving inflammatory arthritis in *DNase II*^−/−^ *STING*^S365A/S365A^ mice downstream of TBK1 recruitment to STING. Blocking these cytokines was shown to improve the symptoms of human arthritis (Choy et al., 2020; Ma and Xu, 2013) but did not fully control the disease, probably due to other cytokines that can also cause inflammation (Brennan and McInnes, 2008). Treatments that can block the upstream STING-TBK1 interaction may provide benefits for self-DNA–induced inflammatory arthritis by inhibiting the production of most if not all inflammatory cytokines.

## Discussion

Previous studies in mouse models with defective DNA clearance revealed a critical role for the cGAS–STING pathway in mediating autoimmunity (Ablasser et al., 2014; Ahn et al., 2012; Gall et al., 2012; Gao et al., 2015; Gray et al., 2015). Excessive type I IFN signaling mediated embryonic lethality in DNase II–deficient mice (Kawane et al., 2006; Yoshida et al., 2005); however, *DNase II*^−/−^ *Ifnar1*^−/−^ mice still developed severe polyarthritis (Kawane et al., 2006). In this study, we used three different *DNase II*^−/−^ *STING* mutant mice that have distinct signaling defects (S365A, L373A, and ΔCTT) to determine the molecular mechanism downstream of STING activation that is necessary for inducing cytokines and inflammatory arthritis. We found that *DNase II*^−/−^ *STING*^S365A/S365A^ mice, but not *DNase II*^−/−^ *STING*^ΔCTT/ΔCTT^ or *DNase II*^−/−^ *STING*^L373A/L373A^ mice, developed polyarthritis characterized by inflammation and bone erosion due to the production of inflammatory cytokines such as TNF-α and IL-6. As the STING-L373A mutation in *DNase II*^−/−^ mice completely abrogated disease phenotypes, these results show that TBK1 recruitment to STING not only mediates IFN-dependent lethality but also drives the IFN-independent inflammatory arthritis caused by defective DNA clearance. The L373A mutation in the TBK1-binding motif of STING may also disrupt IKKε recruitment, because the sequence of TBK1 involved in binding to STING is highly conserved in IKKε. IKKε is an inducible gene, and its expression levels are usually lower than those of TBK1 in most cells. Nevertheless, IKKε and TBK1 function redundantly in activating NF-κB downstream of STING in myeloid cells (Balka et al., 2020). Thus, the observed phenotypes of the STING-L373A mice are likely caused by impairment of both TBK1 and IKKε recruitment; however, this has yet to be demonstrated directly.

Because the STING-L373A and S365A mutants still induce autophagy, our data shows that STING-induced autophagy does not promote or prevent disease pathogenesis during DNase II deficiency. Although STING activation was shown to cause cell death in some cells such as T lymphocytes in an IFN-independent manner (Gaidt et al., 2017; Gulen et al., 2017; Larkin et al., 2017), the contribution of STING-induced cell death in this arthritis model may be minor, as blocking TNF-α alone prevented the development of arthritis in *DNase II*^−/−^ *STING*^S365A/S365A^ mice (Fig. 6, A and B). Therefore, The NF-κB pathway induced by TBK1 recruitment to STING is the most likely candidate for inducing these inflammatory cytokines.

While DNase II degrades DNA in the lysosome, three-prime repair exonuclease 1 (TREX1 or DNase III) degrades DNA in the cytosol (Yang et al., 2007). Loss-of-function mutations of the TREX1 gene caused autoinflammatory diseases such as Aicardi-Goutières syndrome and familial chilblain lupus (Crow et al., 2006; Rice et al., 2015; Richards et al., 2007; Tüngler et al., 2012). TREX1-deficient mice survived until adulthood but developed severe autoinflammatory disease (Morita et al., 2004; Stetson et al., 2008). Similar to *DNase II*^−/−^ mice, disease phenotypes of *Trex1*^−/−^ mice such as mortality and inflammation were rescued by the loss of cGAS or STING (Gall et al., 2012; Gao et al., 2015; Gray et al., 2015). These results suggest that cGAS–STING pathway activation is a major cause of autoinflammatory diseases triggered by self-DNA.

Unlike *DNase II*^−/−^ mice that develop inflammatory arthritis in an IFN-independent manner, *Trex1*^−/−^ mice lacking IRF3 or IFNAR1 were rescued from lethality and inflammation (Stetson et al., 2008). What causes these discrepancies of IFN dependence in these DNase-deficient models? It is conceivable that more cytoplasmic DNA may accumulate in *DNase II*^−/−^ cells than in *Trex1*^−/−^ cells. When cytoplasmic DNA is limited, intracellular cGAS concentrations (i.e., one allele vs. two alleles of the cGAS gene expression) become critical for cGAS activation, because its activation requires DNA-induced liquid phase separation, which depends on the concentrations of both cGAS and DNA (Du and Chen, 2018). *DNase II*^−/−^ mice required the deletion of both alleles of the cGAS gene to rescue mice from lethality, whereas deleting one allele of the cGAS gene was sufficient to largely eliminate disease phenotypes in *Trex1*^−/−^ mice (Gao et al., 2015), suggesting abundant levels of cytoplasmic DNA and stronger activation of the cGAS–STING pathway in *DNase II*^−/−^ mice. Therefore, robust activation of the STING-induced NF-κB pathway may contribute to the disease phenotypes more strongly in *DNase II*^−/−^ mice than in *Trex1*^−/−^ mice. In addition, the types of cells being activated may differ in the absence of DNase II versus TREX1. In DNase II–deficient mice, macrophages are believed to be the major source of inflammation, as they are responsible for engulfing and digesting nuclei from apoptotic cells (Nagata, 2005). The contribution of macrophage-induced inflammation to arthritis remains to be determined in vivo by depleting macrophages or specifically deleting the STING gene in macrophages during DNase II deficiency. In this regard, *DNase II*^−/−^ *STING*^S365A/S365A^ mice, which survive into adulthood and develop arthritis, provide a good model to study the cell types driving the inflammation. The role of neutrophils or monocytes in arthritis also needs to be tested, as the chemoattractant CXCL1 and CXCL2 were up-regulated in *DNase II*^−/−^ *STING*^S365A/S365A^ mouse paws (Fig. 4, A–C). The cell types responsible for the disease phenotypes of *Trex1*^−/−^ mice require further investigation.

cGAS, activated by cytosolic DNA, produces cGAMP that binds to the cytosol-facing cGAMP-binding site of STING. Defects in TREX1 functions lead to the accumulation of cytosolic DNA that can directly bind to cGAS, whereas defects in DNase II result in undigested DNA in lysosomes. Abnormal macrophages carrying undigested DNA were found in the fetal liver and thymus of *DNase II*^−/−^ mice (Kawane et al., 2001; Kawane et al., 2003). How lysosomal DNA reaches the cytosol to activate cGAS is still unclear, but studies have suggested that the lysosome is a vulnerable organelle that can undergo ruptures (Terman et al., 2006). As excessive amounts of DNA accumulate in lysosomes in the absence of DNase II, some lysosomes may undergo rupture and release DNA into the cytosol.

It is surprising that *DNase II*^−/−^ mice do not display any overt phenotypes in the absence of cGAS or STING, suggesting that these mice can tolerate excess DNA that is not digested in the lysosome. One would have expected that the excess cellular DNA could activate other DNA sensors, such as TLR9 and absent in melanoma 2 (AIM2), to cause inflammatory diseases. The embryonic lethality of the *DNase II*^−/−^ mice could not be rescued by deleting the AIM2 gene, although AIM2 deficiency reduced inflammatory arthritis in *DNase II*^−/−^ *Ifnar1*^−/−^ mice (Baum et al., 2015; Jakobs et al., 2015). Ablating TLR9 failed to rescue the lethality of *DNase II*^−/−^ mice (Okabe et al., 2005) and failed to reduce the arthritis of *DNase II*^−/−^ *Ifnar1*^−/−^ mice (Kawane et al., 2006). However, 10-mo-old *DNase II*^−/−^ *Ifnar1*^−/−^ mice have been reported to develop splenomegaly and produce antinuclear antibodies in a STING- or AIM2-independent manner; instead, Unc93b, which regulates the localization of several TLRs (e.g., TLR3, TLR7, and TLR9) to the endosome, is required (Baum et al., 2015; Pawaria et al., 2015). Mice may elicit other signaling pathways including the TLR9 pathway after the long-term presence of lysosomal DNA during *DNase II*^−/−^ deficiency. Further research is needed to determine why defective DNA clearance in the *DNaseII*^−/−^ mice primarily activates cGAS but not AIM2 or TLR9.

In summary, using *DNase II*^−/−^ *STING* mutant mouse models, our study provides the molecular mechanism by which STING activation causes autoinflammatory arthritis during DNase II deficiency. TBK1 recruitment to STING is required not only for IFN signaling that causes embryonic lethality but also for producing inflammatory cytokines that drive arthritis. These data suggest that an inhibitor interfering with TBK1 binding to STING may provide therapeutic benefits for self-DNA–induced inflammatory diseases such as arthritis. Interfering with TBK1 recruitment does not affect STING-induced autophagy but specifically abrogates cytokine production including, but not limited to, type I IFNs, TNF-α, and IL-6. This molecular mechanism may also apply to other STING-dependent autoimmunity. In this regard, a mouse model of STING-associated vasculopathy with onset in infancy that harbors gain-of-function mutations of STING developed inflammation even in the absence of IRF3 (Warner et al., 2017). Further studies on the role and molecular mechanism of STING-driven inflammation will provide new insights into drug development for autoimmune diseases.

## Materials and methods

### Mice and treatments

All mice used in this study were on the C57BL/6 background. *DNase II*^+/−^ mice were from Dr. Shigekazu Nagata (Kyoto University, Kyoto, Japan) and crossed with *IFNAR1*^−/−^ mice from Dr. David Farrar (University of Texas Southwestern Medical Center, Dallas, TX). *STING*^ΔCTT/ΔCTT^, *STING*^L373A/L373A^, and *STING*^S365A/S365A^ mice were generated by the CRISPR/Cas9 technology in our laboratory as previously described (Yum et al., 2021). *STING*^GT/GT^ mice were purchased from The Jackson Laboratory. Genomic DNA from toes or tails was extracted using KAPA Express Extract Kits (Roche) for genotyping. All mice were bred and maintained under specific pathogen–free conditions in the animal facility at the University of Texas Southwestern Medical Center according to experimental protocols approved by the Institutional Animal Care and Use Committee. Mice were not littermate controlled because of the relatively low number of pups in each litter and the need for a large number of mice for the experiments. Wild-type C57BL/6 mice were purchased from The Jackson Laboratory. Mice were gender- and age-matched for all experiments.

InVivoMAb anti–TNF-α (XT3.11), rat IgG (HRPN), and anti–IL-6R (15A7) were from BioXcell. 6- or 5-mo-old *STING*^S365A/S365A^ mice were treated with the indicated amounts of each antibody by i.p. injection once or twice a week as shown in the figure legends. For anti–IL-6R treatment, the mice were first treated with the antibody i.v., followed by i.p. injection once a week.

### Clinical assessment and pathology

The arthritis score of the forelimb and hindlimb joints was assessed manually: 0, no swelling; 1, mild swelling; and 2, severe swelling or deformation of the limb or finger (Kawane et al., 2006). The scores of four limbs were combined to give a total arthritis score for each mouse. For pathology, joint tissues were fixed in 4% (wt/vol) paraformaldehyde, decalcified in 11.4% EDTA, embedded in paraffin, cut into 5-µm sections, and stained with H&E or TRAP. Histologic inflammation and bone erosion scores were measured as described previously (Scales et al., 2016). For inflammatory cell infiltrates, the scoring system was as follows: 0, no infiltrate detected; 1, modest leukocyte infiltration in synovial tissue; 2, moderate leukocyte infiltration in synovial tissue; and 3, gross leukocyte infiltration in synovial membrane with significant loss of synovial and articular architecture. For erosion of cartilage and bone: 0, no abnormalities; 1, fibrillation of cartilage and/or mild erosive infiltration of periosteal and subchondral bone; 2, moderate fibrillation and loss of cartilage and/or moderate erosive infiltration of periosteal and subchondral bone; and 3, significant loss of cartilage and/or erosive infiltration of periosteal and subchondral bone.

TRAP staining was scored on a scale of 0–5 as follows: 0, no TRAP-positive cells; 1, rare TRAP-positive cells in BM or soft tissues; 2, few TRAP-positive cells seen in areas of resorption; 3, moderate TRAP-positive cells at least one site of resorption or attached to periarticular sites without bone erosion; 4, moderate TRAP-positive staining at multiple areas of resorption or within resorption pits; and 5, marked TRAP-positive cells at most or all areas of bone resorption (O’Brien et al., 2016). Images were acquired using an E400 microscope (Nikon) and NES software.

### Quantitative RT-PCR (qRT-PCR)

RNA was isolated from homogenized mouse paws using TRIzol Reagent (Invitrogen). Reverse transcription and qRT-PCR were done using the cDNA reverse transcription kit and SYBR green master mix from Applied Biosystems following the manufacturer’s instructions. The primer sequences used for qRT-PCR are listed in Table 1.

**Table 1.** Mouse qRT-PCR primers

Gene	Forward (5′ to 3′)	Reverse (5′ to 3′)
*GAPDH*	TGG​CAA​AGT​GGA​GAT​TGT​TGC​C	AAG​ATG​GTG​ATG​GGC​TTC​CCG
*TNF-α*	TCC​CAG​GTT​CTC​TTC​AAG​GGA	GGT​GAG​GAG​CAC​GTA​GTC​GG
*IL-6*	TCC​AGT​TGC​CTT​CTT​GGG​AC	GTA​CTC​CAG​AAG​ACC​AGA​GG
*MMP-3*	ACT​CTA​CCA​CTC​AGC​CAA​GG	TCC​AGA​GAG​TTA​GAC​TTG​GTG​G
*CXCL1*	TCC​AGA​GCT​TGA​AGG​TGT​TG	GTC​TGT​CTT​CTT​TCT​CCG​TTA​CTT
*CXCL2*	ATG​CCT​GAA​GAC​CCT​GCC​AAG	GGT​CAG​TTA​GCC​TTG​CCT​TTG

### Flow cytometry and ELISA

Blood was collected into EDTA anticoagulant tubes and stained with various fluorescent dye–conjugated antibodies targeting the following antigens from BioLegend: Ly6G-FITC (1A8), CD115-PE (AFS98), CD11b-PerCP (M1/70), Ly6c-AF647 (HK1.4), CD11c-BV605 (N418), and CD45-AF700 (30-F11). For FACS staining, anti–mouse CD16/32 (BioLegend) was added into 100 µl of anticoagulated blood in a 1:200 ratio and incubated for 10 min. Fluorescent dye–conjugated antibodies were then added to the blood in a 1:200 ratio and incubated at room temperature for 30 min in the dark. 2 ml of 1× RBC lysis solution (349202; Thermo Fisher Scientific) was directly added into the blood/antibody mixture and incubated at room temperature for 10 min. The mixture was then centrifuged at 350 *g* for 5 min, and the supernatant was discarded. The pellet was washed three times with FACS buffer (3% FBS in PBS with 0.5 M EDTA). Stained cells were analyzed using an LSRII (BD Biosciences) and FlowJo. In detail, CD45^+^ cells were gated for CD11b^+^ Ly6G^−^ cells, which were further gated for CD115^+^ cells (monocytes). The CD115^+^ monocyte population was gated for ly6c^+^ monocytes and ly6c^−^ monocytes. We confirmed that the gated CD115^+^ monocyte population did not contain CD11c^+^ cells. The serum levels of TNF-α and IL-6 were determined with ELISA kits (R&D Systems) according to the manufacturer’s instructions.

### Statistical analysis

All statistical analyses were performed with GraphPad Prism 8 software using a two-tailed, unpaired Student’s *t* test. All data are presented as mean ± SD.
